# Molecular Genetic Investigation of Digital Melanoma in Dogs

**DOI:** 10.3390/vetsci9020056

**Published:** 2022-01-30

**Authors:** David Conrad, Alexandra Kehl, Christoph Beitzinger, Thomas Metzler, Katja Steiger, Nicole Pfarr, Konrad Fischer, Robert Klopfleisch, Heike Aupperle-Lellbach

**Affiliations:** 1 Department of Pathology, LABOKLIN GmbH & Co. KG, 97688 Bad Kissingen, Germany; aupperle@laboklin.com; 2 Department of Molecular Biology, LABOKLIN GmbH & Co. KG, 97688 Bad Kissingen, Germany; kehl@laboklin.com (A.K.); beitzinger@laboklin.com (C.B.); 3 Institute of Pathology, School of Medicine, Technische Universität München, 81675 München, Germany; thomas.metzler@tum.de (T.M.); katja.steiger@tum.de (K.S.); nicole.pfarr@tum.de (N.P.); 4 School of Life Sciences Weihenstephan, Technische Universität München, 85354 Freising, Germany; konrad.fischer@tum.de; 5 Department of Pathology, Freie Universität Berlin, 14163 Berlin, Germany; Robert.Klopfleisch@fu-berlin.de

**Keywords:** canine, acral, mutation, tumour, *BRAF*, *KRAS*, *NRAS*, *c-kit*, CNV, *KITLG*

## Abstract

Canine digital melanoma, in contrast to canine oral melanoma, is still largely unexplored at the molecular genetic level. The aim of this study was to detect mutant genes in digital melanoma. Paraffin-embedded samples from 86 canine digital melanomas were examined for the *BRAF* V595E variant by digital droplet PCR (ddPCR), and for exon 11 mutations in *c-kit.* Furthermore, exons 2 and 3 of *KRAS* and *NRAS* were analysed by Sanger sequencing. Copy number variations (CNV) of *KITLG* in genomic DNA were analysed from nine dogs. The *BRAF* V595E variant was absent and in *c-kit*, a single nucleotide polymorphism was found in 16/70 tumours (23%). The number of copies of *KITLG* varied between 4 and 6. *KRAS* exon 2 codons 12 and 13 were mutated in 22/86 (25.6%) of the melanomas examined. Other mutually exclusive *RAS* mutations were found within the hotspot loci, i.e., *KRAS* exon 3 codon 61: 2/55 (3.6%); *NRAS* exon 2 codons 12 and 13: 2/83 (2.4%); and *NRAS* exon 3 codon 61: 9/86 (10.5%). However, no correlation could be established between histological malignancy criteria, survival times and the presence of *RAS* mutations. In summary, canine digital melanoma differs from molecular genetic data of canine oral melanoma and human melanoma, especially regarding the proportion of *RAS* mutations.

## 1. Introduction

Melanomas are malignant neoplasms originating from melanocytes and are often found in the oral cavity, on the skin, at the eyes or the digits [1]. Pathogenesis of canine melanocytic neoplasms depends on the site [1] and the coat colour [2]. Furthermore, occurrence, breed predisposition and prognosis vary depending on the site of melanocytic neoplasms [1]. Cutaneous and ocular melanocytic neoplasms are mainly benign [1,3] and are commonly located at the head, ventral abdomen, scrotum or the eye [1]. In contrast, oral melanomas are highly aggressive, and benign variants are rare [1,4]. Melanocytic neoplasms of the toe have an intermediate aggressiveness: Nishiya et al. [1] stated that they appear to be malignant in 5–58% of cases. Breed predispositions have been described in Schnauzers [5], Irish Setters [5,6], Golden Retrievers [6], Rottweilers and Labrador Retrievers [7].

Various mutations have already been identified in human melanoma and to some extent also in canine melanoma, as explained below. In humans, the *BRAF* V600E variant is well known as a common cause of tumour formation, as it is a T to A transversion in exon 15, resulting in the amino acid substitution of valine by glutamic acid at codon 600 [8,9]. This *BRAF* variant, in the past also referred to as V599E, is detectable in about 60% of human melanomas [10,11,12,13] and activates MEK as a part of the mitogen-activated protein kinase (MAPK) pathway [8]. This variant is of special therapeutic interest, because vemurafenib, an inhibitor specifically targeting the activated step of the MAPK pathway induced by the mutated BRAF protein, is available, and treating human melanoma patients with this drug results in a transient remission of the disease [14,15]. The canine *BRAF* V595E mutation is orthologous to human *BRAF* V600E with a transversion from T to A in exon 15 [9]. This somatic mutation was specifically found in canine urothelial and prostatic carcinoma, but only rarely in other canine tumours [9].

The MAPK pathway is activated by *RAS* genes and RAS proteins (especially KRAS/NRAS/HRAS) are upstream of the RAF proteins within the signal transduction [8]. According to a review by Downward [16], RAS proteins show somatic mutations in human cancer in up to 20% of cases. Point mutations in codons 12, 13 and 61 within the *RAS* genes have already been identified as oncogenes in human cancers [17]. In human melanomas, mutations of *NRAS* (10–15%) and *KRAS* (2%) are detectable, with *NRAS* having the substitution L61Q in about 80% of cases while G12V accounts for about 77% of the *KRAS* mutations according to a review by Cicenas et al. [18]. Molecular genetic studies are also available for human acral melanoma. *NRAS* mutations are frequently found (27.9%), while *HRAS* or *KRAS* mutations are only present in less than 5% of cases [19]. Mayr et al. [20] identified *NRAS* exon 2 codon 61 mutations in 2 of 16 dogs with cutaneous malignant melanoma, located in lumbar and scrotal regions. In contrast to this, Escobar et al. [21] did not find any *KRAS* or *NRAS* mutations in 11 canine malignant melanomas (site not reported). Additionally, *RAS* mutations have been described in two canine cutaneous melanomas (one *KRAS* and one *NRAS*) and in three melanomas of the digits (two *KRAS* and one *NRAS*) [22].

The proto-oncogene *c-kit* encodes the transmembrane receptor tyrosine kinase c-Kit (CD117) [23], while *KITLG* encodes stem cell factor (SCF) as a ligand of the tyrosine-kinase receptor c-Kit [24]. c-Kit is expressed in numerous cell types such as mast cells, melanocytes, and interstitial cells of Cajal [25]. In melanocytes, the proto-oncogene *c-kit* is thought to be involved in the regulation of pigmentation, migration, differentiation, survival, and proliferation [25]. Mutations of this gene have been identified in human mucosal melanoma [26,27,28] and also in human acral melanoma [26,28]. In canine melanoma, gain-of-function *c-kit* mutations have been found in a limited number of cases [29,30,31]. Smedley et al. [30] identified 85 variants in *c-kit* in canine oral melanomas, including 9 non-synonymous mutations.

*KITLG* is physiologically involved in postnatal cutaneous melanogenesis and in the terminal differentiation of follicular epithelial melanocytes, making it significant for the determination of canine coat colour [32]. Karyadi et al. [33] identified a copy number variation (CNV) at the *KITLG* locus, which is likely to be responsible for the high risk of black Poodles to develop digital squamous cell carcinoma (DSCC). They found an increased risk for DSCC in dogs with more than four copies of the *KITLG* gene. Dark-coloured dogs have a well-known predisposition for DSCC, which also shows a more aggressive histological picture in such breeds [34]. *KITLG* mutations have not been investigated in canine melanoma so far.

As it is generally known that there are differences between melanocytic neoplasms of different sites [1], the first oncogenetic studies of canine oral melanoma may not be representative for digital melanoma in dogs. In addition, the number of canine digital melanomas that have been addressed in the available literature is small [5,35,36,37]. Thus, the aim of our retrospective study was to investigate the occurrence of genetic aberrations of *BRAF*, *c-kit*, *KITLG*, *NRAS* and *KRAS* in canine digital melanoma. The hypothesis was that their oncogenic mechanisms may differ from melanomas of other sites, particularly oral ones. Furthermore, the molecular genetic results should be compared to their human counterparts. For this we used formalin-fixed toes with melanomas, which were submitted for routine diagnostics during the years 2014–2021 to Laboklin GmbH & Co. KG, Bad Kissingen, Germany and corresponding blood samples as far as available.

## 2. Materials and Methods

Malignant melanomas of the toe/nailbed from 86 dogs were included in this retrospective study. Inclusion criteria were a clear histopathological diagnosis and the availability of a paraffin-embedded tumour block before decalcification. Melanomas of the skin of the limb which did not affect the nailbed, or the bone were excluded. Age, sex, breed and further information, if available, were collected. For additional information, the veterinarians were contacted by telephone. Coat colour, exact site of the digital mass and a short report of the clinical course were documented whenever possible.

Blood samples of nine dogs with digital melanoma were available from routine diagnostics (presurgical or geriatric screening). As all samples (toes and blood) were submitted for routine diagnostic purposes, ethics committee approval was not required. All the material used was no longer needed for diagnostics.

### 2.1. Histopathology

Claws were trimmed according to Kamstock et al. [38] and decalcified using Osteomoll^®^ rapid decalcifier solution for histology (Merck, Darmstadt, Germany) over a period of 24–72 h. Representative sample sites were embedded in paraffin wax and processed for routine histopathological examination according to standard procedures. From each toe, one non-decalcified tumour site was used for further molecular genetic investigations. Sections were stained with haematoxylin and eosin (HE) and histologically evaluated by trained veterinary pathologists during routine diagnostics and reevaluated by H.A.-L. Bleaching with 30% H_2_O_2_ was performed for 24–72 h, if necessary, to detect nuclear atypia or mitoses if pigmentation was too intensive. Representative sites were characterised according to morphological criteria (pigmentation, mitotic count, nuclear atypia, and histomorphological type) as described by Spangler and Kass [3].

### 2.2. Molecular Genetic Methods

#### 2.2.1. BRAF Mutation, c-kit Gene Mutation

Paraffin-embedded samples were prepared for DNA extraction using the QIAamp^®^ DNA FFPE Tissue Kit (Qiagen, Hilden, Germany) according to the manufacturer’s instructions. Isolated DNA was examined for the presence of the *BRAF* mutation c.1784T > A by digital droplet polymerase chain reaction (ddPCR) using a mutation-specific TaqMan^®^ assay as described by Mochizuki et al. [39]. Analysis was performed using DropletReader (Bio-Rad, Feldkirchen, Germany) and QuantaSoft™ Software (Bio-Rad, Feldkirchen, Germany). The sequence of exon 11 of *c-kit* (ENSCAFT00030034940.1) was analysed by PCR amplification and subsequently by Sanger sequencing. Primers are listed in Table 1. Bidirectional Sanger sequencing of all PCR products was performed on an ABI 3130 Genetic Analyzer (Life Technologies, Carlsbad, CA, USA) using the BigDye Terminator v1.1 Cycle Sequencing Kit (Life Technologies, Carlsbad, CA, USA) according to standard protocols. Mutational screening for *BRAF* and *c-kit* was done at the Department of Molecular Biology, Laboklin GmbH & Co. KG, Bad Kissingen, Germany.

#### 2.2.2. NRAS/KRAS Mutation Analysis

Referring to the genome CanFam3.1, melanoma samples were analysed for mutations in exons 2 and 3 of *NRAS* (ENSCAFT00000015144.4) and *KRAS* (ENSCAFT00000010525.4), respectively, by Sanger sequencing at the Institute of Pathology, Technische Universität München, Germany. DNA was isolated from a microdissected section of a tumour tissue block from areas in which a high tumour cell concentration (at least 60% tumour cell content, median: 80%, range: 60–95%) had been microscopically identified. DNA isolation was performed using the Maxwell 16 RSC extraction system (Promega, Madison, WI, USA). DNA quantity was measured by a QuBit 4.0 system and the QuBit high sensitivity assay (both: Thermo Fisher Scientific, Waltham, MA, USA). All exons were amplified with the primers listed in Table 1 and with 10–20 ng of DNA as input and an annealing temperature of 60 °C. Bidirectional Sanger sequencing of all PCR products was subsequently conducted on an ABI 3100 Genetic Analyzer (Life Technologies, Carlsbad, CA, USA) using the BigDye Terminator v1.1 Cycle Sequencing Kit (Life Technologies, Carlsbad, CA, USA) according to standard protocols.

#### 2.2.3. Copy Number Variation Analysis of KITLG

Genomic DNA was isolated from ethylenediaminetetraacetic acid (EDTA) blood with the MagNA Pure 96 system using a DNA Tissue Lysis Buffer and viral NA Small kit (Roche, Basel, Switzerland) according to the manufacturer’s instructions. Copy number quantification of the *KITLG*-CNV was performed by ddPCR using TaqMan^®^ assays specific for the *KITLG*-CNV sequence and proto-oncogene 1 (ETS1) as reference gene as described by Bannasch et al. [32]. An additional assay was designed for *KITLG*-CNV using the CanFam3.1 reference genome. Primers and the probe are listed in Table 1. The copy number was determined using DropletReader (Bio-Rad, Feldkirchen, Germany) and QuantaSoft™ Software (Bio-Rad, Feldkirchen, Germany). Analysis of the samples was performed at the Department of Molecular Biology, Laboklin GmbH & Co. KG, Bad Kissingen, Germany; with both assays and ETS1 as reference, each in duplicate. Two independent in-house studies consistently demonstrated that both the inter- and intra-assay spearman correlation between the two methods (Bannasch et al. [32] and Karyadi et al. [33]) was highly significant (*p* < 0.001). Therefore, we calculated the median from all four measurements (two per method) to obtain robust CNV estimates.

### 2.3. Statistical Analysis

Statistical significance analyses were evaluated using IBM SPSS Statistics (version 26). Comparisons between *RAS* mutations (*KRAS* and *NRAS* combined) and *KRAS* and the characteristics of degree of pigmentation, mitotic count and degree of nuclear atypia were performed with the Mann-Whitney U test. The association of *RAS*/*KRAS* mutations and histomorphological type was investigated by Fisher’s exact test. The value *p* < 0.05 was considered statistically significant. Survival times were determined using the Kaplan–Meier plot and matched by the logrank test. The significance level was *p* < 0.05, the number of degrees of freedom was 1.

## 3. Results

### 3.1. Case Description, Clinical Data, and Survival Time

Toes from 86 dogs were collected for the study. Clinical data were often incomplete because the animals were only presented to the clinics for toe amputation. As far as available, details are listed in the suppl. Table. The age ranged from five to 15 years, with a median of 11 years. In three cases, the age was unknown. The cohort included 36 intact and 15 castrated male dogs as well as 18 intact and 16 spayed female dogs. Sex was unknown in one case. The following breeds were represented: 23 mongrels, 15 Labrador Retrievers, 10 Giant Schnauzers, seven Rottweilers, four Golden Retrievers, three Irish Terriers, three Sheepdogs (not specified), two Bernese Mountain Dogs, two Cocker Spaniels and two Poodles. There was one animal of each of the following breeds: Airedale Terrier, Belgian Shepherd, Bullmastiff, Cairn Terrier, Cane Corso, Doberman, French Bulldog, German Hunting Terrier, German Shepherd, Gordon Setter, Havanese, Miniature Schnauzer, Pinscher, Scottish Terrier, and a Tibetan Terrier. A large proportion of the animals had a black coat colour (*n* = 31). The others were black & tan (*n* = 15), yellow (*n* = 8), fawn (*n* = 6), brown (*n* = 5), tricolour (*n* = 2) and white (*n* = 2). In 17 cases, the coat colour could not be verified anymore.

Melanomas affected the forelimb (*n* = 54) more often than the hindlimb (*n* = 23). In nine cases, the relevant information could not be retrieved. The further clinical course as well as the current health status could be determined in 66 cases and were unknown in the remaining 20 cases. At the time of data collection, 15 dogs were still alive with a median follow-up time of 137 days (range: 19–639 days). 22 animals died because of progressive tumour disease with associated metastasis. Metastases were not clearly localised (*n* = 11), affected the lungs (*n* = 9), spinal cord (*n* = 1), or the tail (*n* = 1). Another 29 dogs died for other reasons (e.g., cardiac or renal insufficiency). Of all dogs that died, the one-year survival rate was 24% (12 dogs) and the two-year survival rate was 12% (6 dogs). Within the group of animals that died with reported metastasis, three (14%) survived one year and one (5%) survived two years.

### 3.2. Pathological Findings

**Figure 1:**
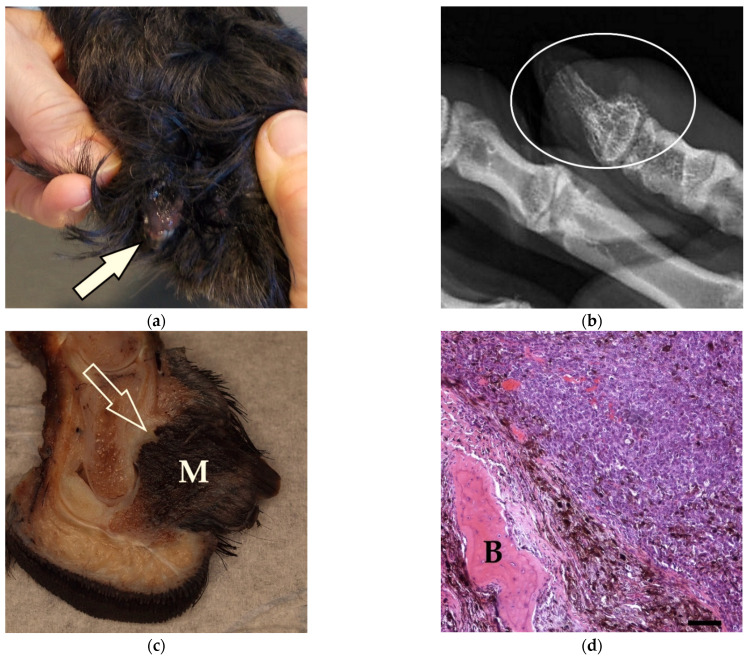
Malignant melanoma of the fifth toe of the hindlimb of a 10-year-old Giant Schnauzer (Dog No. 48): (**a**) The claw horn has detached and the neoplastic tissue is exposed (arrow; © Dr. Blasberg, Kleintierpraxis Bad Honnef); (**b**) The claw bone shows significant osteolysis in the X-ray (circle; © Dr. Blasberg, Kleintierpraxis Bad Honnef); (**c**) Longitudinal cut of the formalin-fixed amputated digit with a 1.4 × 1.0 × 1.4 cm black mass (M) originating from the nailbed and infiltrating the bone (arrow); (**d**) Histologically, the neoplastic cells show varying degrees of pigmentation and infiltration of the bone (B) (HE, bar = 200 µm).

The melanomas of the toes were mostly 0.5–2.0 cm in diameter, but some neoplasms reached a size of up to 5.5 × 5.5 × 5.5 cm. The claw was detached in 40% of cases. Neoplasms affected the nailbed or the paw, and distal phalanges were mostly destructed (Figure 1). The degree of pigmentation was low (*n* = 48), moderate (*n* = 17), intense (*n* = 6) or varied greatly within the neoplasm (*n* = 15) (Figure 1d). In 60 tumours, 2–15 mitotic figures/10 high-power field (HPF) were counted. In 20 tumours, the mitotic count was 21–39/10 HPF and in six cases, the mitotic count was 44–65/10 HPF (Table S1). Nuclear atypia was mild (*n* = 14), moderate (*n* = 48) or severe (*n* = 24). Most melanomas had a dominant epithelioid morphology (*n* = 44). In 14 cases, the tumour cells were mostly spindle-shaped. Round cell morphology (*n* = 9) or balloon cell type (*n* = 1) were rare. In the remaining 18 cases, morphology was a mixture of these types. Margins were clean in 69 dogs, narrow (<3 mm) in seven tumours and infiltrated by tumour cells in 10 cases.

The different histological criteria were statistically analysed with regard to their influence on the median survival time. Dogs with melanomas larger than 1 cm in diameter (median: 151 days; *n* = 11) and those with tumour cells detectable at the surgical margins (median: 72 days; *n* = 4) had lower survival times than animals with smaller melanomas (median: 274 days; *n* = 5) and clean margins (median: 183 days; *n* = 15). However, due to the low number of cases, statistical significance was not reached.

### 3.3. Genetic Analysis

Wild type *BRAF* V595E was detected in 81 samples. In five cases, PCR failed, as not enough DNA could be extracted for amplification.

In 16 of 70 melanomas, we identified a silent *c-kit* gene alteration in exon 11. In 16 other samples, no PCR product could be amplified. The *c-kit* variant in exon 11 was c.1731C > T (counterpart in human molecular pathology: p.Y577Y) and either heterozygous (*n* = 10) or homozygous (*n* = 6). This variant corresponds to a putative germline variant listed in ENSEMBL (SNP-No.: rs853024368; https://www.ensembl.org/index.html, accessed on 22 November 2021).

In *KRAS* and *NRAS* genes, exons 2 and 3 were amplified and sequenced. Most mutations were detected in the *KRAS* gene (Table 2). All identified *RAS* mutations were mutually exclusive—combinations did not occur.

*KRAS* mutations in exon 2 (codons 12 and 13) were found in 22 cases which are listed in Table 3. *KRAS* mutations in exon 3 (codon 61) were found in the mixed cell type melanoma of the third toe of the right forelimb of an eight-year-old male German Shepherd (No. 80; survival time: 55 days) and in one round cell type melanoma of the fifth toe of the right forelimb of an eight-year-old female mongrel (No. 9; survival time: 110 days). The amount of pigments was moderate in the first case and low in the second. The mitotic count was 31/10 HPF in the German Shepherd and 5/10 HPF in the mongrel.

*NRAS* mutations in exon 2 (codons 12 and 13) were detected in digital melanomas of the right forelimbs of two Golden Retrievers. Codon 12 mutation was found in a low pigmented, epithelioid melanoma with 8 mitoses/10 HPF of an eight-year-old male Golden Retriever (No. 56; unknown survival time). Codon 13 mutation was present in a low pigmented, mixed cell type melanoma with five mitoses/10 HPF of a six-year-old female spayed Golden Retriever (No. 57). This dog was still alive 12 months after diagnosis. *NRAS* mutations in exon 3 (codon 61) were found in nine cases which are summarised in Table 4.

Statistical analysis showed that the degree of pigmentation, mitotic count, degree of nuclear atypia or histomorphological type of digital melanomas, as well as the survival time, were neither significantly associated with *RAS* mutations in general nor with *KRAS* mutations. As there were only 11 cases of *NRAS* mutation, statistical analysis was not performed for *NRAS* mutations alone to avoid non-reliable data. Regarding the survival times, an empirical median of 142 days was calculated for *RAS* mutants (*KRAS* and *NRAS*), and 183 days for wild types. However, the difference was not statistically significant.

### 3.4. Copy Number Variations (CNV) of KITLG

EDTA blood samples were available from nine animals and examined for copy number variations (CNV) of the *KITLG* locus. The detected copy numbers varied between four and six (Table 5). Two dogs (Nos. 27 and 73) had copy numbers of five and six, respectively, and showed a mutation in *KRAS* exon 2 codon 12. Furthermore, a 14-year-old Giant Schnauzer (No. 43) had six copies of the *KITLG* gene locus in addition to a mutation in *NRAS* exon 3 codon 61. In two other cases, mutations were found in *NRAS* exon 2 codon 13 (No. 57) and *NRAS* exon 3 codon 61 (No. 11), combined with a CNV of four (Table 5). 

## 4. Discussion

In this study, the goal was to identify various somatic mutations in canine digital melanoma and to compare these with other canine melanomas, especially oral melanoma. Additionally, the correlation between the mutations and histological criteria of malignancy was investigated. Furthermore, CNV of *KITLG* were analysed as a germline mutation. The most striking result was the high proportion of *KRAS* mutations in canine digital melanoma and an increased copy number of *KITLG* (4–6) was observed. A statistically significant correlation between detected mutations and histomorphological criteria of malignancy and survival time was not found.

Melanoma is a genetically complex disease and the molecular context of *BRAF*, *RAS* and *c-kit* gene mutation may affect the response to targeted treatment and even immunology-based therapies in man [40,41]. This is the first study investigating various mutations in a larger number of digital melanomas in dogs. Until now, only few molecular genetic analyses have been performed to compare canine melanomas to their human counterparts [2,42,43,44,45]. Moreover, these studies were limited by their retrospective characterisation of cases without complete clinical and pathological data.

In general, the number of canine digital melanomas reported in the literature is rather low (Wobeser et al. *n* = 52 [35]; Marino et al. *n* = 24 [36]; Schultheiss *n* = 27 [37]; Henry et al. *n* = 10 [5]). However, in a study on 2912 digital lesions in dogs [7], our working group identified 196 malignant melanomas. None of these studies used molecular methods to examine the digital melanomas. Hendricks et al. [22] analysed *RAS*, *BRAF* and *c-kit* gene mutations, among others, in three digital melanomas, and Chu et al. [29] investigated *c-kit* mutation in three digital melanomas. It is well known that canine melanocytic neoplasms show very different clinical and pathological features at different sites [1,3]. Thus, the hypothesis of the present study was that canine digital melanomas may have different oncogenetic mechanisms than, for instance, oral melanomas. Human acral melanomas have also been studied at the genetic level, with mutations of *BRAF* (21.3%) and *KIT* genes (11.5%; 50% within exon 11) standing out. Here, too, *NRAS* mutations in codons 12, 13 and 61 were found (27.9%), but only less than 5% of cases had activating mutations in *HRAS* or *KRAS* [19]. The mutational analysis of the present study was therefore based on the data on human melanoma and studies on canine, mostly oral, melanoma.

Within the canine digital melanomas in our study, we found no *BRAF* V595E variant. Hendricks et al. [22] did not find any *BRAF* mutations in 37 canine melanomas—including three originating from the digits. No *BRAF* mutation was detected in canine oral melanoma (Gillard et al. *n* = 77 [2]; Shelly et al. *n* = 17 [46]). In contrast, Mochizuki et al. [39] found one *BRAF* mutation in a canine cutaneous melanoma and two in mucosal melanomas, studying a total of 54 melanomas. Thus, mutations in *BRAF* seem to not play a relevant role in the pathogenesis of digital melanoma in dogs.

In our screening of canine digital melanomas for *c-kit* mutations, we only found a common polymorphism whose frequency in the dog population is reported to be 18% (https://www.ebi.ac.uk/eva/?eva-study=PRJEB24066, accessed on 25 November 2020). Comparison with past studies is only possible to a limited extent, because there are currently only a few studies available that have investigated the presence of c-*kit* mutations in canine melanoma. In those studies, digital melanomas were not represented at all [2,30,45,47,48], or they were clearly underrepresented [29]. In summary, *c-kit* mutations may occur in canine melanoma [29,30], but seem to play a minor role. However, no *c-kit* mutation was detected in digital melanoma in previous studies [29]. Since only tumour tissue and no corresponding normal tissue was examined in our studies, it is not possible to verify whether a *c-kit* mutation is of somatic origin or a germline mutation. As we only detected silent alterations in our samples, it was not further investigated and may be clarified in future studies.

To the best of our knowledge, this is the first investigation of copy number variations of *KITLG* in dogs affected by melanoma. The number of copies varied between four and six in the nine dogs from which blood samples were available for genetic analysis. Mutations in *KITLG* have been identified as a factor in the oncogenesis of melanoma in man [49]. Germline mutations relevant for tumour predispositions have already been identified in dogs [44]. It is important to keep in mind that a germline mutation may reflect a genetic predisposition rather than the direct somatic oncogenetic pathway like *BRAF* or *c-kit* mutations. Bannasch et al. [32] found that CNV of *KITLG* are linked to a variation in the dog’s coat colour and pointed out that *KITLG* is an important factor in melanogenesis. The median number of copies varied from two to eight, depending on the breed and the coat colour [32]. Furthermore, CNV at the *KITLG* locus have been detected in dogs diagnosed with canine digital squamous cell carcinoma and seem to be involved in its development [33]. In our study, five of nine dogs examined for CNV of *KITLG* had a dark coat colour (*n* = 4 black; *n* = 1 black & tan) and four of these dogs had more than four copies. Within the remaining four of nine dogs (coat colour: *n* = 2 fawn; *n* = 1 yellow; *n* = 1 unknown), only two dogs were found with more than four copies. Black-coated breeds such as Beauce Shepherds [2], Rottweilers or Schnauzers [2,50] appeared to be predisposed to malignant melanoma in any site while pale-coated dogs, especially solid white-haired breeds, were underrepresented [2]. This is also reflected by the animals in our study, in which most dogs were black (*n* = 31) or black & tan (*n* = 15) and only 23 dogs were light coloured. As discussed by Grassinger et al. [7], the high number of Giant and Standard Schnauzers in our data set is due to their German origin.

Based on these data, we hypothesise that CNV of *KITLG* may be involved in the development of melanoma in dogs with dark and black coats. However, the lack of control data makes interpretation of the results difficult. Thus, at the moment, it is impossible to give any breeding recommendations to minimise the risk of digital melanoma in certain dog breeds.

In three canine digital melanomas, Hendricks et al. [22] detected two cases of *KRAS* mutations and one *NRAS* mutation. In our study, a much larger sample size was processed: Overall, RAS mutations were detected in 40.7% of the tumours examined. *KRAS* exon 2 codons 12 and 13 mutation were detected in 22 of 86 dogs (25.6%) and *KRAS* exon 3 codon 61 mutation in two of 55 cases (3.6%). *NRAS* exon 2 codons 12 and 13 were mutated in two of 83 cases (2.4%), and *NRAS* exon 3 codon 61 mutation was found in nine of 86 cases (10.5%). These results may indicate that *RAS* mutation could be a driver of mutation in canine digital melanoma. Interestingly, these mutations were mutually exclusive, and we did not identify an additional somatic aberration in any other investigated gene (*BRAF*, *c-kit*).

A comparison to canine oral melanoma was also sought. Hendricks et al. [22] reported *KRAS* mutation rates of 3% in canine mucosal melanomas. Wong et al. [45] identified this mutation in 5% of all canine oral melanomas examined in their study. All variants affected the previously described hotspot loci. This supports our hypothesis that canine digital melanomas differ from oral (and mucosal) melanomas at the genetic level, especially regarding the clear difference of detectable *KRAS* mutations.

Since HRAS alterations are relatively rare in human tumours [16], we did not include this gene in our studies. However, this may be part of future research as our recent findings showed differences between human and canine digital melanoma.

In human medicine, promising results are already available in the field of RAS-targeted treatment of melanomas [14]. There are similar approaches for dogs as well. Wei et al. [51] investigated the effect of dual MEK and PI3K/mTOR inhibition on canine melanoma cell lines with ERK and AKT/mTOR activation, and were able to inhibit tumour growth. The study also looked for NRAS mutations, which, however, only occurred in small numbers. Their presence or absence did not seem to be related to ERK activity. Fowles et al. [52] took a similar approach, examining dual inhibition of MAPK and PI3K/AKT in human and canine melanoma and reporting detectable inhibition in both species. They also discussed mutation-independent MAPK and PI3K/AKT activation. Further research in this area is already in progress and seems to be necessary.

The mutations found were examined regarding their diagnostic and prognostic relevance. It is well known that histological criteria, such as mitotic count or degree of pigmentation, can be used to differentiate between benign and malignant melanocytic neoplasms [53]. However, histological criteria, immunohistochemical expression patterns and other prognostic factors were not clearly correlated to survival time in dogs with digital melanomas. Furthermore, in our study, there was no statistically significant difference in the histological criteria (pigmentation, mitotic count, nuclear atypia, and histomorphological type) between dogs with or without *RAS* mutation. The same applied to the comparison of the *KRAS* mutant with the *KRAS* wild type. Thus, *KRAS* exon 2 and 3 mutations in canine digital melanomas probably have no prognostic relevance. Unfortunately, there were not enough cases with *NRAS* exon 2 and 3 mutations (*n* = 11) to perform reliable statistics.

According to the literature, 32% of dogs with malignant digital melanoma had metastases at presentation and another 26% developed metastases after surgery [36]. Henry et al. [5] reported a metastasis rate of 40% at the time of diagnosis. Dogs with digital malignant melanoma were reported to have a one-year survival rate of 42% to 44% and a two-year survival rate of 11% to 13%, which reflects the high metastatic rate of the tumour [5,36].

The survival rate of all dogs, including those in which the melanoma diagnosis could not be determined as the confirmed cause of death, was 24% for one year and 12% for two years. The one- and two-year survival rates were 14% and 5%, respectively, within the group that died of melanoma, i.e., already had metastasis at the time of death. The survival rates determined in this study show a similar trend to previous literature [5,36] and confirm the poor prognosis of canine digital melanoma.

The present study was limited by its retrospective nature, where clinical data and exact survival times could only be requested retrospectively and were thus partly incomplete. Future studies could collect this information in a timely manner and make the data more precise. Thus, the statistical analysis of the diagnostic and prognostic relevance of *RAS* mutations was partly not feasible without producing unreliable data. Whole genome sequencing of digital melanoma in dogs would also be attractive, allowing even more potential genetic mutations to be investigated.

In summary, our initial hypothesis was that canine digital melanoma may differ in its molecular genetic pathway from oral melanoma. This hypothesis was accepted, since we found significantly more *KRAS* mutations in canine digital melanoma compared to data reported for oral melanoma. Furthermore, a difference is also obvious between canine and human acral melanoma, in which *BRAF* and *KIT* mutations occur more frequently.

## 5. Conclusions

It is already known that malignancy and prognosis of canine melanoma strongly depend on the site of the tumour. The present study showed that canine digital melanoma differs from oral melanoma at the molecular genetic level as well. Mutations of the *KRAS* gene are especially common. In contrast, mutations of *BRAF* and *c-kit* genes which are involved in human melanoma obviously do not play a role in the oncogenesis of canine digital melanoma.

## Figures and Tables

**Table 1:** Primer/Probe sequences used for the analysis of *NRAS*, *KRAS*, *c-kit* and *KITLG* genes.

Gene	Primer/Probe	Sequence
*NRAS* exon 2	Forward Reverse	5′-CGCCCATTAAACCTAATTGC-3′ 5′-ACCAAAAGCCAGAGGTAGGG-3′
*NRAS* exon 3	Forward Reverse	5′-ATCTCCTACCCTCCACACCC-3′ 5′-GGCAAATACACAGAGGAAGCC-3′
*KRAS* exon 2	Forward Reverse	5′-AAAAGGTGTTGATAGAGTGGGTTATAC-3′ 5′-AAATGGGCCTGCACAAATC-3′
*KRAS* exon 3	Forward Reverse	5′-ACTGTGTTTCTCCCTTCTCAGG-3′ 5′-GCCCTCGATGTCATTTTATTATATTC-3′
*c-kit* exon 11	Forward Reverse	5’-CCCATGTATGAAGTACAGTGGAAG-3′ 5′-GTTCCCTAAAGTCATTGTTACACG-3′
CNV *KITLG*	Forward Reverse Probe	5′-TGCACAAGGGAGAAGGGTTG-3′ 5′-AGATGGTCCTGGGGAAACCA-3′ 5′-FAM-TGGCTGGGGACAGAAGCAATG-BBQ650-3′

**Table 2:** Overview of all cases with detected *RAS* mutation (*n* = 35).

Gene	Mutation	Wild Type	Not Done
*KRAS* exon 2 codon 12	17	64	0
*KRAS* exon 2 codon 13	5		
*KRAS* exon 3 codon 61	2	53	31
*NRAS* exon 2 codon 12	1	81	3
*NRAS* exon 2 codon 13	1		
*NRAS* exon 3 codon 61	9	77	0

**Table 3:** Characteristics of dogs with *KRAS* exon 2 mutation (codons 12 and 13) in digital melanoma (*n* = 22).

Dog No.	Codon	Breed (Coat Colour)	Age (y)	Sex	Toe	Morphology	Pigment (Degree)	Mitoses/ 10 HPF	Survival Time (d)
40	12	GS	11	MC	RF	mixed	2	7	157
39	12	GS	8	M	LF	epithelioid	2	3	72
25	12	LR	12	M	RH	epithelioid	1	13	338
26	12	LR (U)	12	F	LF	spindle cell	1	4	274
27	12	LR (black)	14	M	RF	epithelioid	1	5	A
24	12	LR (yellow)	10	FS	RF	epithelioid	1	5	1304
49	12	RW	8	F	RF	epithelioid	1	24	U
77	12	DM	U	F	LF	mixed	2	5	U
75	12	CT	11	MC	LF	epithelioid	V	7	1016
73	12	BS	9	M	LH	epithelioid	1	13	A
1	12	MG (black)	13	M	LF	mixed	1	58	U
2	12	MG (U)	8	MC	RF	epithelioid	1	25	40
3	12	MG (U)	10	F	LF	round	1	15	142
4	12	MG (U)	9	FS	RF	mixed	2	22	U
5	12	MG (U)	9	M	RH	epithelioid	3	3	1079
6	12	MG (U)	13	M	U	round	1	22	A
7	12	MG (b&t)	9	F	LF	epithelioid	3	4	189
8	13	MG (U)	10	MC	RH	epithelioid	V	9	A
50	13	RW	5	F	U	epithelioid	1	65	194
51	13	RW	11	M	LH	epithelioid	1	15	18
28	13	LR (black)	12	M	LH	epithelioid	1	9	504
82	13	Havanese	15	M	LH	round	1	23	U

Abbreviations: y: years; d: days; M: male; MC: male castrated; F: female; FS: female spayed; RF: right forelimb; RH: right hindlimb; LF: left forelimb; LH: left hindlimb; U: unknown; A: alive; V: varying; b&t: black and tan; BS: Belgian Shepherd; CT: Cairn Terrier; DM: Doberman; GS: Giant Schnauzer; LR: Labrador Retriever; MG: mongrel; RW: Rottweiler.

**Table 4:** Characteristics of dogs with *NRAS* exon 3 mutation (codon 61) in digital melanoma (*n* = 9).

Dog No.	Breed (Coat Colour)	Age (y)	Sex	Toe	Morphology	Pigment (Degree)	Mitoses/ 10 HPF	Survival Time (d)
41	GS	9	M	RH	epithelioid	1	34	83
42	GS	10	MC	LF	epithelioid	3	6	52
43	GS	14	M	U	epithelioid	V	21	U
66	BMD	8	M	LH	spindle cell	2	4	U
63	Sheepdog	9	M	LF	round	V	9	48
70	Poodle	11	M	RF	mixed	3	4	U
68	CS	11	FS	U	spindle cell	2	5	U
10	MG (b&t)	14	M	LF	mixed	2	3	840
11	MG (U)	8	MC	RF	epithelioid	3	3	A

Abbreviations: y: years; d: days; M: male; MC: male castrated; FS: female spayed; RF: right forelimb; RH: right hindlimb; LF: left forelimb; LH: left hindlimb; U: unknown; A: alive; V: varying; b&t: black and tan; BMD: Bernese Mountain Dog; CS: Cocker Spaniel; GS: Giant Schnauzer; MG: mongrel.

**Table 5:** Characteristics of dogs analysed for CNV of *KITLG* (*n* = 9).

Dog No.	Copy Number	*RAS* Mutation	Breed (Coat Colour)	Age (y)	Sex	Toe	Morphology	Pigment (Degree)	Mitoses/ 10 HPF	Survival Time (d)
43	6	*NRAS* exon 3 codon 61	GS	14	M	U	epithelioid	V	21	U
48	5	-	GS	10	M	RH	epithelioid	2	2	A
73	5	*KRAS* exon 2 codon 12	BS	9	M	LH	epithelioid	1	13	A
57	4	*NRAS* exon 2 codon 13	GR	6	FS	RF	mixed	1	5	A
27	6	*KRAS* exon 2 codon 12	LR (black)	14	M	RF	epithelioid	1	5	A
61	5	-	IT	13	M	LF	epithelioid	0	3	A
11	4	*NRAS* exon 3 codon 61	MG (U)	8	MC	RF	epithelioid	3	3	A
22	5	-	MG (b&t)	15	M	LH	spindle cell	1	15	A
21	4	-	MG (black)	8	F	RF	round	1	15	A

Abbreviations: y: years; d: days; M: male; MC: male castrated; F: female; FS: female spayed; RF: right forelimb; RH: right hindlimb; LF: left forelimb; LH: left hindlimb; U: unknown; A: alive; V: varying; b&t: black and tan; BS: Belgian Shepherd; GR: Golden Retriever; GS: Giant Schnauzer; IT: Irish Terrier; LR: Labrador Retriever; MG: mongrel.

## Data Availability

Data available upon request due to privacy/ethical restrictions.

## Supplementary Materials

The following supporting information can be downloaded at: https://www.mdpi.com/article/10.3390/vetsci9020056/s1. Table S1: Case description, clinical and histomorphological findings of dogs with digital melanoma (*n* = 86).
