# Supplementary material for: Molecular Genetic Investigation of Digital Melanoma in Dogs
Source: Vet Sci. 2022 Jan 30;9(2):56. doi: 10.3390/vetsci9020056 (PMC8874500; doi:10.3390/vetsci9020056)
Supplement: Supplementary file 1 [file vetsci-09-00056-s001.zip › vetsci-1520508-supplementary.pdf]

veterinary sciences

MDPI

Molecular Genetic Investigation of Digital Melanoma in Dogs

David Conrad [1,*], Alexandra Kehl [2], Christoph Beitzinger [2], Thomas Metzler [3], Katja Steiger [3], Nicole Pfarr [3], Konrad Fischer [4], Robert Klopfleisch [5] and Heike Aupperle-Lellbach [1]

Table S1. Case description, clinical and histomorphological findings of dogs with digital melanoma (*n* = 86).

**Dog No.**	**Breed**	**Age (y)**	**Sex** M: male intact MC: male castrated F: female intact FS: female spayed	**Phenotypical haircoat colour**	**Affected digit** RF: right forelimb RH: right hindlimb LF: left forelimb LH: left hindlimb	**Affected toe**	**Reported metastasis**	**Degree of pigmentation** L: low M: moderate I: intense V: varying	**Mitoses/ 10 HPF**	**Nuclear atypia** MI: mild MO: moderate S: severe	**Cell morphology** E: epithelioid S: spindle shaped R: round B: balloon M: mixed	**Margins** C: clean I: infiltrated N: narrow
1	Mongrel	13	M	black	LF	U		L	58	MI	M	C
2	Mongrel	8	MC	U	RF	U		L	25	MI	E	C
3	Mongrel	10	F	U	LF	2nd	Yes	L	15	MO	R	C
4	Mongrel	9	FS	U	RF	3rd		M	22	S	M	I
5	Mongrel	9	M	U	RH	U		I	3	MI	E	C
6	Mongrel	13	M	U	U	U		L	22	S	R	C
7	Mongrel	9	F	black & tan	LF	U		I	4	MO	E	C
8	Mongrel	10	MC	U	RH	U		V	9	MO	E	C
9	Mongrel	8	F	black	RF	5th	Yes	L	5	S	R	C
10	Mongrel	14	M	black & tan	LF	2nd	Yes	M	3	MO	M	C
11	Mongrel	8	MC	U	RF	3rd		I	3	MI	E	C
12	Mongrel	13	FS	black	RH	U		M	10	MO	E	N
13	Mongrel	U	F	black	LF	U		L	36	MI	E	C

14	Mongrel	10	F	U	LF	2nd	Yes	V	27	MO	E	C
15	Mongrel	11	M	black	RF	2nd	Yes	V	57	MO	E	C
16	Mongrel	11	FS	black	RF	U		L	5	MO	S	C
17	Mongrel	12	MC	U	LH	U		V	3	MO	R	C
18	Mongrel	10	F	black	RF	3rd		L	12	MI	S	C
19	Mongrel	11	FS	black	RH	U		L	9	S	E	C
20	Mongrel	10	M	U	RH	5th		L	7	S	S	C
21	Mongrel	8	F	black	RF	3rd		L	15	MI	R	C
22	Mongrel	15	M	black & tan	LH	5th		L	15	MO	S	C
23	Mongrel	11	F	brown	RF	U		L	39	MO	S	C
24	Labrador Retriever	10	FS	yellow	RF	3rd		L	5	MO	E	C
25	Labrador Retriever	12	M	brown	RH	4th	Yes	L	13	S	E	C
26	Labrador Retriever	12	F	U	LF	3rd	Yes	L	4	MO	S	I
27	Labrador Retriever	14	M	black	RF	3rd		L	5	MO	E	C
28	Labrador Retriever	12	M	black	LH	5th	Yes	L	9	MO	E	N
29	Labrador Retriever	8	M	black	RH	4th		L	6	MO	E	C
30	Labrador Retriever	11	MC	yellow	LF	U		L	3	MI	B	C
31	Labrador Retriever	13	MC	black	LF	U	Yes	L	26	MI	S	C
32	Labrador Retriever	11	M	yellow	LH	U		L	10	MO	E	C
33	Labrador Retriever	10	F	U	RH	U	Yes	V	23	MO	S	C
34	Labrador Retriever	12	FS	black	RF	3rd		V	13	MO	M	N

35	Labrador Retriever	12	M	U	RF	U		V	28	S	M	C
36	Labrador Retriever	12	M	brown	U	U	Yes	I	3	MI	E	C
37	Labrador Retriever	10	MC	brown	U	U		V	5	MO	R	C
38	Labrador Retriever	14	FS	brown	RF	2nd		L	25	S	E	C
39	Giant Schnauzer	8	M	black	LF	5th	Yes	M	3	MO	E	I
40	Giant Schnauzer	11	MC	black	RF	U		M	7	MO	M	I
41	Giant Schnauzer	9	M	black	RH	4th		L	34	MO	E	C
42	Giant Schnauzer	10	MC	black	LF	3rd	Yes	I	6	MO	E	I
43	Giant Schnauzer	14	M	black	U	U	Yes	V	21	S	E	C
44	Giant Schnauzer	10	MC	black	LF	2nd		L	9	S	E	N
45	Giant Schnauzer	7	FS	black	RF	1st		M	5	MO	E	C
46	Giant Schnauzer	5	M	black	LF	2nd		V	15	MI	M	C
47	Giant Schnauzer	6	MC	black	RF	3rd		L	34	MO	E	C
48	Giant Schnauzer	10	M	black	RH	5th		M	2	S	E	C
49	Rottweiler	8	F	black & tan	RF	1st		L	24	MO	E	I
50	Rottweiler	5	F	black & tan	U	U	Yes	L	65	S	E	C
51	Rottweiler	11	M	black & tan	LH	2nd	Yes	L	15	S	E	C
52	Rottweiler	9	M	black & tan	RF	1st		L	37	S	E	C
53	Rottweiler	6	F	black & tan	RF	2nd		L	11	MO	S	C

54	Rottweiler	12	U	black & tan	LF	U		M	9	MO	E	C
55	Rottweiler	7	M	black & tan	RH	3rd	Yes	L	5	S	E	N
56	Golden Retriever	8	M	yellow	RF	1st		L	8	MO	E	C
57	Golden Retriever	6	FS	yellow	RF	3rd		L	5	MO	M	C
58	Golden Retriever	12	F	yellow	LH	U		L	44	MO	M	C
59	Golden Retriever	15	FS	yellow	RF	5th		L	7	MI	S	C
60	Irish Terrier	14	F	fawn	RF	U		V	34	MO	R	C
61	Irish Terrier	13	M	fawn	LF	U		L	3	S	E	C
62	Irish Terrier	11	MC	fawn	RF	2nd		M	7	MO	M	N
63	Sheepdog	9	M	white	LF	U		V	9	S	R	C
64	Sheepdog	U	M	black	U	U	Yes	M	13	S	E	I
65	Sheepdog	11	FS	white	LH	4th	Yes	L	28	MO	M	C
66	Bernese Mountain Dog	8	M	tricolour	LH	1st		M	4	MO	S	C
67	Bernese Mountain Dog	11	FS	tricolour	RF	2nd		L	5	S	E	C
68	Cocker Spaniel	11	FS	black	U	U		M	5	MO	S	C
69	Cocker Spaniel	12	FS	black	LF	3rd		L	53	S	M	C
70	Poodle	11	M	yellow	RF	5th		I	4	MO	M	C
71	Poodle	12	F	U	U	U		L	28	MI	S	C
72	Airedale Terrier	6	FS	black & tan	U	U		L	6	MI	S	C
73	Belgian Shepherd	9	M	fawn	LH	4th		L	13	MO	E	C
74	Bullmastiff	10	F	fawn	LF	U		V	54	MO	E	N

75	Cairn Terrier	11	MC	U	LF	U		V	7	MO	E	C
76	Cane Corso	7	M	fawn	LH	5th	Yes	M	9	S	E	C
77	Doberman	U	F	black & tan	LF	U		M	5	MO	M	C
78	French Bulldog	12	M	black	LF	4th	Yes	L	15	S	E	C
79	German Hunting Terrier	13	MC	black & tan	LF	2nd		L	7	MO	M	C
80	German Shepherd	8	M	U	RF	3rd		M	31	S	M	C
81	Gordon Setter	13	M	black & tan	LF	5th		L	5	MO	E	I
82	Havanese	15	M	black	LH	5th		L	23	MO	R	C
83	Miniature Schnauzer	13	M	black	RF	1st		L	7	MO	M	C
84	Pinscher	12	MC	black & tan	LF	2nd		V	13	S	E	I
85	Scottish Terrier	10	FS	black	RH	1st		M	7	MO	M	I
86	Tibetan Terrier	11	M	U	RF	3rd	Yes	M	4	MO	E	C

U: unknown
